# Towards Accurate and Reliable ICU Outcome Prediction: A Multimodal Learning Framework Based on Belief Function Theory using Structured EHRs and Free-Text Notes

**DOI:** 10.1007/s41666-025-00219-3

**Published:** 2025-11-30

**Authors:** Yucheng Ruan, Daniel J. Tan, See-Kiong Ng, Ling Huang, Mengling Feng

**Affiliations:** 1 Saw Swee Hock School of Public Health, National University of Singapore, Singapore, Singapore; 2 Institute of Data Science, National University of Singapore, Singapore, Singapore; 3 Institute of Clinical Sciences, Imperial College London, London, UK

**Keywords:** Multimodal learning, Belief function theory, Evidence fusion, ICU outcome prediction, Electronic health records

## Abstract

Accurate Intensive Care Unit (ICU) outcome prediction is critical for improving patient treatment quality and ICU resource allocation. Existing research mainly focuses on structured data, e.g. demographics and vital signs, and lacks effective frameworks to integrate clinical notes from heterogeneous electronic health records (EHRs). This study aims to explore a multimodal framework based on belief function theory that can effectively fuse heterogeneous structured EHRs and free-text notes for accurate and reliable ICU outcome prediction. The fusion strategy accounts for prediction uncertainty within each modality and conflicts between multimodal data. Experiments on two large ICU datasets demonstrate that our method achieves superior predictive performance compared to existing approaches. For example, it improving F1 score and AUPRC by 6.51% and 3.72%, respectively, and increases predictive reliability with an 18.08% decrease in Brier score for mortality prediction in MIMIC-III dataset. Comparable improvements are consistently observed on the ZICIP dataset, underscoring the predictability and reliability of the approach. These improvements translate into fewer false positives, supporting more precise triage decisions and more efficient allocation of critical care resources. Beyond ICU outcome prediction, the proposed framework offers a versatile tool for multimodal EHR analysis, with potential applications across diverse clinical tasks.

## Introduction

Intensive Care Unit (ICU) is a specialized hospital ward that offers comprehensive and continuous care to critically ill patients. As the population of critically ill patients grows, the demand for ICUs has risen significantly, placing strain on already limited and costly intensive care resources [1], especially during public health crises like the COVID-19 pandemic, when hospitals face an overwhelming surge of patients [2].

Due to the limited availability of intensive care resources, researchers have emphasized the necessity of predicting ICU outcomes such as mortality rates and prolonged lengths of stay (PLOS). Accurate predictions can help in the efficient allocation of medical resources for patients in need and reduce unnecessary expenses without compromising patient care. Furthermore, they are crucial for healthcare providers in making informed decisions about patient care strategies and providing early interventions to patients at high risk of adverse outcomes [3, 4].

Over the past two decades, the adoption of electronic ICU technology has enabled the collection of extensive data on ICU patients, creating new opportunities for developing advanced methods to predict ICU outcomes. Most previous research has concentrated on modeling ICU outcome predictions using structured EHR data [5–7], which often captures only a portion of clinical information. It may miss out on the rich contextual information that unstructured EHR data (such as nursing notes, patient narratives, and imaging reports) can provide. Natural language processing (NLP) techniques have been well explored to extract valuable insights from unstructured free-text EHR data [8–11]. Therefore, effective multimodal learning algorithms are essential to integrate heterogeneous EHRs for better ICU outcome prediction.

Recently, deep learning-based multimodal models have been proven to combine structured EHR data and free-text data at the deep feature level for clinical outcome predictions [12–14]. These models often simply concatenate structured data with encoded features from free texts to generate patient representations for decision-making. While those approaches have improved prediction accuracy, the clinical impacts between the two heterogeneous modalities [15] are now well explored. Another limitation of existing research is the lack of reliability evaluation for deep learning models. Unreliable predictions can lead to incorrect diagnoses or treatment plans, potentially harming patients [16–18]. Therefore, evaluating the predictive reliability is crucial beyond just predictive accuracy, especially in critical care settings. However, concerns about the reliability of existing models in noisy and unstable clinical environments still remain.

Belief function theory (BFT), also known as Dempster-Shafer theory (DST), is a powerful framework for modeling, reasoning with, and integrating imperfect (noisy, uncertain, conflicting) data [19–21]. The effectiveness of BFT in low-quality and multimodal medical image analysis has been widely reviewed in [22, 23]. However, the study of BFT in EHR data is limited. Ling et al. [24] first studied the survival prediction uncertainty using structured EHRs under the framework of BFT and possibility theory. The heterogeneity of clinical data and other medical modalities, e.g., imaging and genetic, are also studied in [25] by combining multimodal data using the generated Dempster’s combination rule [26].

In this work^1^, we further study the effectiveness of BFT in multimodal EHR analysis using structured EHRs and free-text notes with a focus on ICU outcomes prediction. We propose a multimodal learning model under the BFT framework with accurate and reliable ICU outcomes prediction using multimodal EHR data. Instead of developing more effective feature extraction or interaction strategies for multimodal data communication, our framework focuses on effective evidence fusion study and integrates information based on the evidence derived from different modalities. Specifically, we use state-of-the-art deep neural networks for single-modality feature extraction: ResNet/Transformer-based models for structured EHR data and pre-trained language models for free-text EHR data. The extracted features are independently mapped into evidence with an evidence mapping module and then combined in the evidence space in an evidence fusion module. Experimental results on two large-scale EHR datasets, MIMIC-III and ZICIP, for mortality and prolonged length of stay (PLOS) predictions demonstrate the effectiveness of our proposed model in both predictive accuracy and reliability. The code implementation is available on https://github.com/yuchengruan/multimodal-bft-icu.

## Preliminaries

### Belief Function Theory

Belief function theory (BFT) was first introduced by Dempster and Shafer [19, 20]. The expressive capabilities of belief functions enable a more accurate representation of evidence than relying solely on probabilities. Let $\Omega = \{\omega _1, \omega _2, \cdots , \omega _M\}$ be a finite set of hypotheses about some question, called the *frame of discernment*. Evidence about a variable taking values in $\Omega$ can be represented by a *mass function*: $2^{\Omega }$ to [0,1] such that $m(\emptyset ) = 0$ and

1 $$\begin{aligned} \sum _{A \subseteq \Omega }m(A) = 1. \end{aligned}$$

For any hypothesis $A \subseteq \Omega$, the quantity *m*(*A*) is interpreted as a share of a unit mass of belief allocated to the hypothesis that the truth is in *A*, and which cannot be allocated to any strict subset of *A* based on the available evidence. Set *A* is called a *focal set* of *m* if $m(A) > 0$. A mass function is said to be Bayesian if its focal sets are singletons, and logical if it has only one focal set. Two mass functions $m_1$ and $m_2$ representing independent items of evidence can be combined conjunctively by *Dempster’s combination rule* [19] $\oplus$ as

2 $$\begin{aligned} (m_{1} \oplus m_{2}) (A) = \frac{\sum _{B \cap C = A} m_1 (B) m_2 (C) }{1-\sum _{B \cap C = \emptyset } m_1 (B) m_2 (C)}, \end{aligned}$$

for all $A \ne \emptyset$, where $\sum _{B \cap C = \emptyset } m_1 (B) m_2 (C)$ is the degree of conflict among the two pieces of evidence, The nice information fusion attribute of BFT points out the high potential in heterogenetic medical data analysis.

After aggregating all available evidence, the final decision of BFT can be made based on the pignistic transformation proposed by Smets in the Transferable Belief Model [28] that combinese all mass functions using the following expression:

3 $$\begin{aligned} p(\omega ) = \sum _{A \subseteq \Omega : \omega \in A} \frac{m(A)}{|A|}, \forall \omega \in \Omega . \end{aligned}$$

### Evidential Neural Network

Denœux [21] proposed an evidential neural network (ENN) that maps imperfect (uncertain, imprecise, or noise) input features into degrees of belief and ignorance (uncertainty) under the framework of BFT [19, 29]. The essential concept of ENN is to consider each prototype as a piece of evidence, which is discounted based on its distance from the input vector. The evidence from different prototypes is then aggregated using Dempster’s combination rule.

**Fig. 1:**
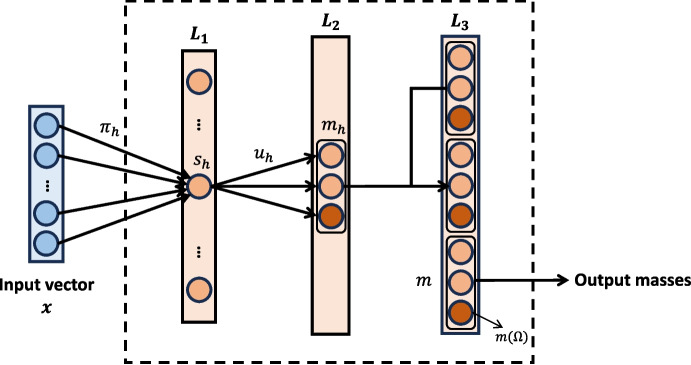
The illustration of ENN

As illustrated in Fig. 1, the ENN consists of one input layer, one hidden layer, and one output layer. The input layer is composed of *H* units (*H* is the number of prototypes), whose weights vectors are prototypes $\pi _{1}, \pi _{2}, \cdots , \pi _{H}$ in input space. The activation of unit *h* in the input layer is

4 $$\begin{aligned} s_{h} = \beta _{h} \textrm{exp}(- \gamma _{h} d_{h}^2), \end{aligned}$$

where $d_{h} = ||x - \pi _{h} ||$ denotes the Euclidean distance between input vector *x* and prototype $\pi _{h}$ , $\gamma _{h} > 0$ is a scale parameter, and $\beta _{h} \in [0, 1]$ is an extra parameter. The hidden layer computes mass functions $m_{h}$ (evidence) of each prototype $\pi _{h}$ is defined as:

5a $$\begin{aligned} m_{h} (\{ \omega _c\})&= u_{h}^{(c)} s_{h}, \quad c = 1, 2, \cdots , M,\end{aligned}$$

5b $$\begin{aligned} m_{h} (\Omega )&= 1 - s_{h}, \end{aligned}$$

where $u_{h}^{(c)}$ is the membership degree of prototype *h* to class $\omega _c$, $\sum _{c = 1}^M u_{h}^{(c)} = 1$, and *M* is the number of classes. Therefore, the vector of mass functions induced by prototypes is denoted as:

6 $$\begin{aligned} m_{h} = (m_{h} (\{ \omega _1\}), m_{h} (\{ \omega _2\}), \cdots , m_{h} (\{ \omega _M\}), m_{h} (\Omega )) \in \mathbb {R}^{M+1}. \end{aligned}$$

Finally, the mass functions are then aggregated by Dempster’s combination rule using (2) in the output layer. A combined mass function *m* is computed as the orthogonal sum of the *H* mass functions:

7 $$\begin{aligned} m = m_{1} \oplus m_{2} \oplus \cdots \oplus m_{H} \in \mathbb {R}^{M+1}. \end{aligned}$$

The combined mass functions (the outputs of the ENN) represent the degrees of belief about the given class with $m(\{ \omega _c\})$, as well as its prediction uncertainty with $m(\Omega )$. In our binary classification case, the dimension of ENN outputs would be three.

## Methods

### Model Architecture

**Fig. 2:**
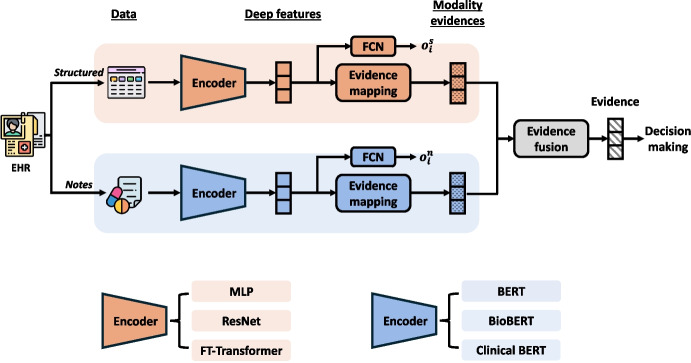
The overview of our proposed framework. EM: evidence mapping and EF: evidence fusion

The overview of the proposed framework is illustrated in Fig. 2. The general idea of this framework is to generate the modality-level evidence for both structured data and free-text notes using the evidence mapping module and fuse the modality-level evidence with Dempster’s combination rule for final prediction.

#### Evidence mapping (EM)

Inspired by ENN and its promising adoptions in medical data analysis [30–33], we propose incorporating ENN as an evidence mapping module with the state-of-the-art encoders to generate evidence for structured EHRs and free-text notes. Given modality level input, the evidence mapping module can output the evidence for each class as well as the uncertainty regarding this prediction.

##### Structured Data Evidence Mapping

To produce modality evidence for structured data, we initially used a structured data encoder to extract deep features (the output dimension is set to 32). We considered three popular encoders to extract the embeddings: MLP, ResNet, FT-Transformer [34] (see Section 4.4 for more details). Subsequently, we introduced an evidence mapping module (the number of prototypes is set to 20) to transform the deep features into evidence embeddings for structured data.

##### Free-Text Notes Evidence Mapping

Similarly, we utilized pre-trained language models to extract the deep features from clinical notes, on which we developed an evidence mapping module (the number of prototypes is set to 20) to produce the evidence of the modality. Our primary analysis focused on pre-trained architectures similar to BERT. Accordingly, three BERT-based architectures were evaluated: BERT [35], BioBERT [35], Clinical BERT [36] (see Section 4.4 for more details). To minimize computational overhead while maintaining high predictive performance, we froze the pre-trained language models and fine-tuned an additional layer (128 hidden units) on top in model training.

#### Evidence Fusion (EF)

Based on the modality-level evidence obtained from structured EHR and free-text notes, we developed the evidence fusion module based on Dempster’s combination rule to generate the final evidence for decision-making.

To combine multiple mass functions $m_1, m_2, \cdots , m_K$ from different modalities/data types/data sources, Dempster’s combination rule is applied again (2) to aggregate evidence for multiple sources for final evidence generation.

8 $$\begin{aligned} m = m_{1} \oplus m_{2} \oplus \cdots \oplus m_{K} \in \mathbb {R}^{M+1}, \end{aligned}$$

where *K* is the number of mass functions to combine. For example, *K*=2 for the fusion of evidence from structured EHRs and free-text notes.

### Augmented Model Optimization Algorithm

We optimize the proposed framework using an augmented learning algorithm, which includes two types of optimization objectives: (1) main objective and (2) auxiliary objective. The main objective is to optimize predictive performance based on transformed evidence as the primary loss function. Additionally, two auxiliary cross-entropy losses are incorporated to enhance the feature representation capability of the independent encoders for the two modalities, as the evidence mapping module performs more effectively with high-quality representations.

Let $p_i = (p_i(\omega _1), \cdots , p_i (\omega _c), \cdots , p_i (\omega _M))$ be the final probability after the pignistic transformation (3) for training sample *i*, and $y_i = (y_{i, 1}, y_{i, 2}, \cdots , y_{i, M})$ denotes the one-hot encoding for corresponding ground-truth labels. The main loss function $\mathcal {L}_{main}$ is computed as:

9 $$\begin{aligned} \mathcal {L}_{main} = - \frac{1}{N} \sum _{i=1}^{N} \sum _{c=1}^{M} w_c y_{i, c} \textrm{log} (p_i (\omega _c)), \end{aligned}$$

where *N* is the number of training samples, *M* denotes the number of classes, and $w_c$ is the weight assigned to each class to address the class imbalance issue.

Moreover, two auxiliary cross-entropy losses are introduced to optimize the feature representation performance of the encoders, as the evidence mapping module performs more effectively with high-quality representations. Firstly, to regulate the representation generated by encoders, we added an additional fully connected network (FCN) to generate logits $o_{i}$ for each modality. Let $o^s_{i} = (o_{i, 1}^{s}, o_{i, 2}^{s}, \cdots , o_{i, M}^{s})$ and $o^n_{i} = (o_{i, 1}^{n}, o_{i, 2}^{n}, \cdots , o_{i, M}^{n})$ be the logits from the encoders for structured data and free-text notes, respectively, the cross-entropy losses $\mathcal {L}^s_{aux}$ and $\mathcal {L}^n_{aux}$ are then calculated with $y_i$ for structured data and notes, respectively:

10 $$\begin{aligned} \mathcal {L}^s_{aux} = - \frac{1}{N} \sum _{i=1}^{N} \sum _{c=1}^{M} w_c y_{i, c} \textrm{log} \frac{\textrm{exp}(o_{i, c}^{s})}{\sum _{b=1}^{M} \textrm{exp}(o_{i, b}^{s})}, \end{aligned}$$

11 $$\begin{aligned} \mathcal {L}^n_{aux} = - \frac{1}{N} \sum _{i=1}^{N} \sum _{c=1}^{M} w_c y_{i, c} \textrm{log} \frac{\textrm{exp}(o_{i, c}^{n})}{\sum _{b=1}^{M} \textrm{exp}(o_{i, b}^{n})}, \end{aligned}$$

Ultimately, the overall loss function $\mathcal {L}_{overall}$ is defined as follows:

12 $$\begin{aligned} \mathcal {L}_{overall} = \mathcal {L}_{main} + \alpha \mathcal {L}^s_{aux} + \beta \mathcal {L}^n_{aux}, \end{aligned}$$

where $\alpha , \beta$ are the hyperparameters that control the balance between the main loss and the auxiliary cross-entropy losses. In both tasks, we set $\alpha = 2$ and $\beta = 1$.

## Experiments

### Datasets

In this study, we selected two large-scale multimodal EHR datasets, MIMIC-III and ZICIP, from different centers for evaluation.

#### MIMIC-III

MIMIC-III (Medical Information Mart for Intensive Care III) is a large, publicly available database containing de-identified health records from patients in critical care units at Beth Israel Deaconess Medical Center (from the US) between 2001 and 2012 [37]. We collected structured data and free-text clinical notes from the database. Patients were excluded if they (1) were under 18 years of age at admission and (2) had incomplete length of stay or mortality data. For patients with multiple ICU stays, we considered only the first.

#### ZICIP

ZICIP (Zigong Intensive Care Infection Patients) is a retrospective single-center database [38] comprising EHRs from adult ICU patients diagnosed with infections at Zigong Fourth People’s Hospital, Sichuan, China, between January 2019 and December 2020. Using the same extraction procedure as for MIMIC-III, we obtained structured data and clinical notes for our study.

### Input Features

Input features include both structured data and unstructured free-text notes in EHRs. In this section, we demonstrate the patient features in our study and provide details about the data preprocessing steps.

#### Structured Data

The structured data were collected during patients’ ICU stays and included demographic information, vital signs and laboratory tests, medical treatments, and comorbidities. For demographic information, the patient’s age, gender, weight, ethnicity, and admission type at the time of admission were included in the MIMIC-III dataset, while only age and gender were available in the ZICIP dataset. Vital signs/lab tests are the most crucial health indicators, easily measured using non-invasive equipment, and are readily understood by all healthcare professionals. For each variable considered, we used the first value recorded within 24 hours of admission time. We then excluded any variables with $\ge 50\%$ missingness rate; this resulted in the inclusion of only heart rate among vital signs features alongside 19 lab test features in MIMIC-III dataset, such as blood urea nitrogen, eosinophil count, and lymphocyte count, over the same period. All vital sign/lab test features were numerical variables. Medical treatments, which include services and interventions provided to patients and recorded in digital systems, were also analyzed. Treatments such as sedatives, statins, diuretics, antibiotics, ventilation, and vasopressors were included, with each treatment feature coded as a binary variable, indicating whether the patient received the treatment. Comorbidities refer to the presence of additional medical conditions, which play a role in decision-making models. In this study, comorbidities such as hypertension, diabetes, alcohol abuse, cerebrovascular accident (stroke), congestive heart failure, and ischemic heart disease were included, all represented as binary variables.

All categorical features were encoded using one-hot encoding, and numerical features were normalized. Missing data were addressed by imputing the median for continuous features and the mode for categorical features, ensuring data consistency. Eventually, the structured data contained 37 features in MIMIC-III dataset and 36 features in ZICIP dataset.

#### Free-Text Notes

Free-text notes contain a rich repository of clinical information about observations, assessments, and the overall clinical picture, which structured data often fails to capture. Furthermore, they provide an important context for interpreting structured data. For instance, while lab results may indicate abnormal values, free-text notes can clarify the relevance of these results by considering the patient’s history, comorbidities, or specific circumstances at the time of testing. As a result, NLP techniques, especially pre-trained LLM, can be applied to these notes to gain deeper insights for data-driven predictions. In MIMIC-III dataset, we focus on *Nursing*, *Nursing/Other*, *Physician*, and *Radiology* notes, as these comprise the majority of clinical documentation and are frequently recorded in the MIMIC-III database [12]. In ZICIP dataset, only *Nursing* notes were available. We extracted only the first 24 hours of notes for each admission to facilitate early outcome prediction.

All notes were preprocessed by appending the feature name at the front to help the pre-trained language model better understand the clinical texts. For instance, if the content *[x]* of a note is under *Nursing*, the processed note would be *Nursing: [x]*. The four types of notes were then concatenated using a newline symbol (\n) to form a unified *Notes* for each patient in the MIMIC-III dataset. Tokenizers from pre-trained language models in Huggingface were employed to break the notes into tokens, standardizing the free-text data for further NLP tasks. The *Notes* were transformed to a fixed length of 512 tokens to ensure input consistency; longer notes were truncated, while shorter notes were padded.

Free-text notes in both datasets have been de-identified in accordance with the Health Insurance Portability and Accountability Act (HIPAA). All protected health information (PHI) has been removed, and access to the dataset requires completion of a data use agreement and ethical training (CITI program).

### Prediction Tasks

In this study, we focus on two ICU prediction tasks: mortality and prolonged length of stay. Since the two clinical tasks are rare in patient popularity, we applied a simple class weighting approach during training based on relative class frequencies to mitigate biases and handle the imbalance in EHR data.

#### Mortality

Mortality is widely acknowledged as a critical task in ICUs. The primary objective of this task is to determine whether a patient is likely to die during their hospital stay. Accurate predictions enable the early identification of high-risk patients and support the efficient allocation of ICU resources. This prediction task is typically framed as a binary classification problem, with the label indicating the occurrence or absence of a death event.

#### Prolonged Length of Stay

Length of stay refers to the duration between a patient’s admission to and discharge from the ICU. In this study, we aim to predict prolonged length of stay (PLOS), defined as a stay exceeding 7 days [12, 39, 40]. Prolonged ICU stays are often linked to severe illnesses, complications, and increased mortality. Moreover, they place considerable pressure on hospital resources by reducing the availability of ICU beds and specialized personnel. Efficient management of ICU LOS not only improves patient outcomes but also enhances the overall effectiveness of healthcare systems. This problem is framed as a binary classification task.

### Baselines

To comprehensively evaluate the effectiveness of our proposed fusion framework, we compared it against three baseline model categories: (1) models using only structured data, (2) models using only free-text notes, and (3) existing multimodal models that integrate both data types.

#### Structured Data Baseline

The following models were used to evaluate performance with structured EHR data:

- Random Forest [41]: A decision tree-based ensemble learning method for making predictions.
- MLP [34]: A fundamental neural network with fully connected layers to encode structured data and serves as a reliable baseline. The model was configured with 3 layers, 32 hidden units, and a dropout rate of 0.1.
- ResNet [34]: Because of the success of ResNet in computer vision [42], it has also been adapted for structured data modeling. Specifically, the main building block is simplified by providing a direct path from input to output. The configuration in our study has 3 residual blocks, 32 hidden units, and a dropout rate of 0.1.
- FT-Transformer [34]: It converts all categorical and numerical features into embeddings, which are then processed through a couple of Transformer layers. It has demonstrated superior performance as a structured data encoder across various tasks. The model configuration has 3 Transformer layers with 192 hidden units, 8 attention heads, and a dropout rate of 0.2.

#### Free-Text Notes Baseline

To assess text-based prediction performance, we compared our model against three BERT-based text classification approaches:

- BERT [35]: A pre-trained language model trained on a large English corpus using self-supervised learning. It learns contextual representations through masked language modeling and next-sentence prediction. In this study, we used the Google-bert/bert-base-uncased model from Huggingface [43] for feature extraction.
- BioBERT [44]: a variant of BERT pre-trained on biomedical literature, such as PubMed abstracts, and is optimized to perform more effectively on biomedical NLP tasks. In this study, we used *dmis-lab/biobert-v1.1* from Huggingface as the extraction model.
- Clinical BERT [36]: A fine-tuned version of BERT on clinical notes from the MIMIC-III database, making it well-suited for handling medical terminology and clinical narratives to enhance performance on clinical tasks. In our study, we used *emilyalsentzer/Bio_ClinicalBERT* from the Huggingface transformer library for extracting embeddings from clinical notes.

#### Multimodal Modal Baseline

To compare against multimodal models, we implemented the concatenation-based approach from [14]. This method combines structured EHR data with extracted text embeddings from free-text notes, followed by two fully connected layers for prediction. To ensure fairness, we tested this approach with BERT, BioBERT, and Clinical BERT as text encoders.

### Implementation Details

The dataset was randomly divided with 60% for training, 20% for validation, and 20% for testing. For model training, a mini-batch size of 32 was used, and the maximum number of epochs was set to 150, with early stopping applied. To handle data imbalance, we used a simple class weighting technique^2^ based on class frequencies, as this was not the main focus of our research.

A 5-fold stratified cross-validation was conducted, and the average performance metrics, along with their standard errors and statistical significance tests, were reported to ensure fair comparisons. Hyperparameters were optimized for all baseline models to achieve the best results. All compared models were implemented using Scikit-learn [45], PyTorch [46], and Hugging Face’s Transformers library [43] in Python 3.8.19. The MLP, ResNet, and FT-Transformer models were built using the original source code on GitHub^3^. The Multimodal approach was modified based on the code on Github^4^.

### Model Evaluation

For comprehensive model evaluation and comparison, we reported the two types of metrics: predictive accuracy and reliability.

- **Predictive accuracy** ensures that models correctly identify critically ill patients who require urgent intervention, thereby reducing the risk of misdiagnosis and unnecessary treatments. To comprehensively assess accuracy, we consider two aspects:
  - **Class-specific** accuracy metrics which evaluate performance separately for positive and negative cases using metrics such as precision, recall, specificity, and negative predictive value (NPV).
    13 $$\begin{aligned} \text {Precision}&= \frac{\text {TP}}{\text {TP} + \text {FP}}, \end{aligned}$$
    14 $$\begin{aligned} \text {Recall}&= \frac{\text {TP}}{\text {TP} + \text {FN}}, \end{aligned}$$
    15 $$\begin{aligned} \text {Specificity}&= \frac{\text {TN}}{\text {TN} + \text {FP}}, \end{aligned}$$
    16 $$\begin{aligned} \text {NPV}&= \frac{\text {TN}}{\text {TN} + \text {FN}}, \end{aligned}$$
    where TP, TN, FP, and FN are True Positive, True Negative, False Positive, and False Negative, respectively.
  - **Holistic** accuracy metrics include balanced accuracy (BACC), F1 score, the area under the receiver operating characteristic curve (AUROC), and the area under the precision-recall curve (AUPRC). AUROC is determined by calculating the area under the ROC curve (TP rate against FP rate across different threshold settings) and AUPRC calculates the area under the Precision-Recall curve across various threshold settings.
    17 $$\begin{aligned} \text {BACC}&= \frac{1}{2} \left( \text {Recall} + \text {Specificity} \right) , \end{aligned}$$
    18 $$\begin{aligned} \text {F1}&= 2 \cdot \frac{\text {Precision} \cdot \text {Recall}}{\text {Precision} + \text {Recall}}. \end{aligned}$$
    BACC, F1 score, and AUPRC are well-suited for evaluating model performance in imbalanced class distributions, as they provide a more balanced assessment than traditional accuracy measures.
- **Predictive reliability** metrics quantify the model’s confidence in predictions. A model with high reliability provides well-calibrated probability estimates, allowing clinicians to make informed risk assessments. Here, we evaluate the performance of the Brier score and negative log-likelihood (NLL).
  19 $$\begin{aligned} \text {Brier Score}&= \frac{1}{N} \sum _{i=1}^{N} (p_i - o_i)^2, \end{aligned}$$
  20 $$\begin{aligned} \text {NLL}&= -\frac{1}{N} \sum _{i=1}^{N} \left( o_i \log (p_i) + (1 - o_i) \log (1 - p_i) \right) , \end{aligned}$$
  where *N* is the number of instances, $p_i$ is the predicted probability of the positive class for instance *i*, and $o_i$ is the actual outcome for instance *i* (1 if positive, 0 if negative).

## Results

### Study Cohorts

Table 1 presents the descriptive characteristics of patients in the MIMIC-III dataset. A total of 38,469 patients met the inclusion criteria, of whom 4,540 (11.8%) died during their stay, and 5,220 (13.6%) experienced a prolonged length of stay. The patient demographics indicate that the majority were White, and there were slightly more male patients than female. Most ICU admissions were classified as emergency cases. Among the patients who died, 51.2% received mechanical ventilation during their ICU stay. Hypertension and congestive heart failure were among the most common comorbidities across the cohort. Additional details on the patient cohort from the ZICIP dataset are provided in Appendix B.

**Table 1:** Characteristics of structured features in the MIMIC-III patient cohort

Patient	Mortality		PLOS	
characteristic	Yes	No	Yes	No
	(N=4540)	(N=33928)	(N=5220)	(N=33248)
Age	68.6 (15.1)	61.6 (16.9)	62.9 (16.3)	62.3 (16.9)
Gender				
Male	2399 (52.8%)	19378 (57.1%)	2943 (56.4%)	18834 (56.6%)
Female	2141 (47.2%)	14550 (42.9%)	2277 (43.6%)	14414 (43.4%)
Weight	79.1 (22.7)	83.0 (23.1)	84.6 (24.3)	82.2 (22.9)
Ethnicity				
White	3100 (68.3%)	24343 (71.7%)	3661 (70.1%)	23782 (71.5%)
Black	260 (5.7%)	2688 (7.9%)	354 (6.8%)	2594 (7.8%)
Asian	106 (2.3%)	1166 (3.4%)	153 (2.9%)	1101 (3.3%)
Hispanic	88 (1.9%)	803 (2.4%)	104 (2.0%)	805 (2.4%)
Other	986 (21.7%)	4928 (14.5%)	948 (18.2%)	4966 (14.9%)
Admission type				
Emergency	4240 (93.4%)	27062 (79.8%)	4480 (85.8%)	26822 (80.7%)
Elective	165 (3.6%)	5911 (17.4%)	533 (10.2%)	5543 (16.7%)
Urgent	135 (3.0%)	955 (2.8%)	207 (4.0%)	883 (2.7%)
Heart rate	91.4 (22.0)	86.8 (18.7)	91.5 (21.0)	86.7 (18.8)
APTT	39.7 (27.1)	34.7 (21.6)	37.2 (23.8)	35.0 (22.2)
BUN	34.1 (25.1)	23.8 (19.0)	28.1 (22.3)	24.5 (19.7)
Eosinophil	1.3 (3.1)	1.5 (1.9)	1.2 (1.7)	1.5 (2.1)
Lymphocytes	12.1 (11.9)	15.5 (11.4)	12.5 (10.8)	15.4 (11.6)
Neutrophils	77.3 (17.7)	76.6 (13.9)	77.8 (15.2)	76.5 (14.4)
RDW	15.5 (2.4)	14.5 (1.9)	14.9 (2.1)	14.6 (2.0)
Bicarbonate	22.6 (5.7)	24.3 (4.4)	23.6 (5.2)	24.1 (4.5)
Chloride	103.3 (7.3)	104.1 (6.0)	104.0 (6.7)	104.0 (6.1)
Creatinine	1.6 (1.4)	1.3 (1.4)	1.4 (1.5)	1.3 (1.4)
Hemoglobin	11.3 (2.3)	11.8 (2.3)	11.6 (2.2)	11.7 (2.3)
Mean cell volume	91.5 (7.7)	89.4 (6.6)	90.3 (7.1)	89.6 (6.7)
Platelet count	227.2 (132.2)	239.0 (112.3)	231.8 (119.8)	238.5 (114.0)
Potassium	4.3 (0.9)	4.2 (0.7)	4.2 (0.8)	4.2 (0.7)
Sodium	138.2 (6.2)	138.6 (4.6)	138.7 (5.2)	138.5 (4.7)
PT	17.4 (10.6)	15.1 (6.8)	16.0 (8.2)	15.3 (7.3)
INR	1.8 (2.2)	1.4 (1.3)	1.6 (1.9)	1.4 (1.4)
WBC	14.3 (16.5)	11.5 (8.8)	12.9 (8.8)	11.6 (10.2)
PLR	39.9 (56.7)	30.4 (47.4)	38.4 (57.5)	30.6 (47.3)
NLR	13.9 (15.6)	9.9 (13.7)	13.0 (15.2)	10.1 (13.8)
Sedatives	1263 (27.8%)	9114 (26.9%)	2360 (45.2%)	8017 (24.1%)
Statin	405 (8.9%)	5164 (15.2%)	556 (10.7%)	5013 (15.1%)
Diuretic	567 (12.5%)	5435 (16.0%)	865 (16.6%)	5137 (15.5%)
Antibiotics	952 (21.0%)	4836 (14.3%)	1122 (21.5%)	4666 (14.0%)
Ventilation	2326 (51.2%)	10923 (32.2%)	3105 (59.5%)	10144 (30.5%)
Vasopressor	1538 (33.9%)	6340 (18.7%)	1639 (31.4%)	6239 (18.8%)
Hypertension	1760 (38.8%)	16221 (47.8%)	2199 (42.1%)	15782 (47.5%)
Diabetes	1107 (24.4%)	9249 (27.3%)	1401 (26.8%)	8955 (26.9%)
Alcohol abuse	202 (4.4%)	1554 (4.6%)	333 (6.4%)	1423 (4.3%)
CVA	309 (6.8%)	1139 (3.4%)	364 (7.0%)	1084 (3.3%)
CHF	1416 (31.2%)	8711 (25.7%)	1867 (35.8%)	8260 (24.8%)
IHD	1276 (28.1%)	12390 (36.5%)	1634 (31.3%)	12032 (36.2%)

For categorical features, the number of instances in each category is reported along with the percentage. For continuous features, the mean and standard deviation are reported in the study

**Table 2:** Comparisons between our best models and clinical scoring baselines in mortality prediction

Dataset	Model	BACC$\uparrow$	F1$\uparrow$	AUROC$\uparrow$	AUPRC$\uparrow$
MIMIC-III	APACHE III	0.5494±0.0016	0.1818±0.0050	0.7823±0.0054	0.4007±0.0099
	SOFA	0.5014±0.0005	0.0057±0.0019	0.6469±0.0033	0.2014±0.0049
	Ours	**0.7658±0.0024**	**0.4498±0.0072**	**0.8515±0.0030**	**0.4819±0.0075**
ZICIP	APACHE III	0.5060±0.0039	0.0242±0.0148	0.5858±0.0195	0.1127±0.0118
	SOFA	0.5077±0.0058	0.0327±0.0217	0.6158±0.0197	0.1048±0.0074
	Ours	**0.6532±0.0116**	**0.5520±0.0186**	**0.7034±0.0117**	**0.5897±0.0153**

### Overall Predictive Performance

In this section, we compared our models with baselines in terms of predictive accuracy and reliability.

#### Predictive Accuracy

To evaluate predictive accuracy, we compared our models with machine learning (ML) baselines. Since clinical scoring systems are widely used by healthcare professionals for mortality risk assessment, we also benchmarked our models against these clinical scoring baselines to highlight their practical relevance.

##### Comparisons with Clinical Scoring Methods

Table 2 presents a comparison between our proposed model and two established scoring methods: APACHE III [47] and SOFA [48]. Our model achieved AUPRC scores of 0.4819 on the MIMIC-III dataset and 0.5897 on the ZICIP dataset, surpassing both clinical scoring systems.

Both APACHE III and SOFA are essentially weighted sums of abnormal physiological values determined through clinical expert consensus. However, their fixed thresholds are not adapted to the underlying data distribution, especially in hight imbalanced datasets.

##### Comparisons with Learning Methods

Table 3 presents the comparison of model performance for holistic predictive accuracy in mortality and PLOS prediction tasks in the MIMIC-III dataset. In the mortality prediction task, our framework, incorporating FT-Transformer and BERT as backbones, achieved the highest F1 score (0.4514). Furthermore, with FT-Transformer and Clinical BERT, it achieved the highest BACC of 0.7658, AUROC of 0.8515, and AUPRC of 0.4819. Compared to the best baseline models, our framework improved predictive performance by approximately 0.75% in BACC, 6.51% in F1 score, 0.66% in AUROC, and 3.72% in AUPRC. For the PLOS prediction task, our framework achieved the highest performance across all evaluation metrics, with a BACC of 0.7017, F1 score of 0.4009, AUROC of 0.7702, and AUPRC of 0.3546. These results represent improvements over the best baselines of 0.72% in BACC, 5.70% in F1 score, 0.84% in AUROC, and 4.42% in AUPRC. Similar patterns were observed in the ZICIP dataset, with further details discussed in Appendix C.

Tables 10 and 11 (Appendix) further demonstrate that the improvements in AUPRC achieved by our proposed models over the baselines are statistically significant. In the ZICIP dataset, the experimental results showed higher standard deviations, likely due to its smaller sample size compared with MIMIC-III. Across both datasets, multimodal approaches consistently outperformed models relying solely on free-text notes. Among the single-modality models, FT-Transformer achieved the best performance.

**Table 3:** Comparison of predictive accuracy on MIMIC-III dataset

Model	Struct.	Notes	BACC$\uparrow$	F1$\uparrow$	AUROC$\uparrow$	AUPRC$\uparrow$
(a) Mortality prediction						
Random Forest	x		0.7128±0.0036	0.3752±0.0038	0.7924±0.0047	0.3620±0.0092
MLP	x		0.7493±0.0018	0.4036±0.0026	0.8311±0.0026	0.4345±0.0068
ResNet	x		0.7524±0.0023	0.4122±0.0026	0.8336±0.0032	0.4385±0.0073
FT-Transformer	x		0.7613±0.0034	0.4179±0.0058	0.8437±0.0033	0.4596±0.0082
BERT as text encoder						
Text encoder only		x	0.6291±0.0029	0.2834±0.0026	0.6794±0.0045	0.2161±0.0045
Multimodal	x	x	**0.7586±0.0030**	0.4130±0.0062	0.8427±0.0029	0.4513±0.0041
Ours (MLP)	x	x	0.7497±0.0048	0.4368±0.0055	0.8362±0.0031	0.4527±0.0057
Ours (ResNet)	x	x	0.7493±0.0032	0.4354±0.0035	0.8376±0.0031	0.4553±0.0080
Ours (FT-Trans)	x	x	0.7557±0.0048		**0.8463±0.0025**	**0.4672±0.0071**
BioBERT as text encoder						
Text encoder only		x	0.6264±0.0048	0.2813±0.0039	0.6812±0.0050	0.2273±0.0029
Multimodal	x	x	**0.7601±0.0028**	0.4170±0.0049	0.8402±0.0031	0.4502±0.0057
Ours (MLP)	x	x	0.7487±0.0043	0.4418±0.0070	0.8378±0.0041	0.4524±0.0070
Ours (ResNet)	x	x	0.7526±0.0045	0.4288±0.0089	0.8397±0.0040	0.4551±0.0080
Ours (FT-Trans)	x	x	0.7581±0.0020	**0.4512±0.0051**	**0.8462±0.0030**	**0.4716±0.0091**
Clinical BERT as text encoder						
Text encoder only		x	0.6554±0.0055	0.3073±0.0050	0.7273±0.0055	0.2760±0.0094
Multimodal	x	x	0.7657±0.0033	0.4238±0.0058	0.8459±0.0034	0.4646±0.0073
Ours (MLP)	x	x	0.7520±0.0051	0.4449±0.0037	0.8428±0.0036	0.4663±0.0065
Ours (ResNet)	x	x	0.7584±0.0049	0.4431±0.0065	0.8466±0.0042	0.4753±0.0081
Ours (FT-Trans)	x	x		**0.4498±0.0072**		
(b) PLOS prediction						
Random Forest	x		0.6734±0.0043	0.3758±0.0042	0.7354±0.0035	0.3128±0.0061
MLP	x		0.6837±0.0012	0.3747±0.0011	0.7487±0.0028	0.3334±0.0053
ResNet	x		0.6849±0.0026	0.3772±0.0033	0.7484±0.0015	0.3299±0.0024
FT-Transformer	x		0.6967±0.0033	0.3793±0.0025	0.7638±0.0033	0.3396±0.0040
BERT as text encoder						
Text encoder only		x	0.6231±0.0189	0.2941±0.0017	0.6434±0.0031	0.2191±0.0042
Multimodal	x	x	0.6892±0.0011	0.3776±0.0021	0.7533±0.0009	0.3342±0.0042
Ours (MLP)	x	x	0.6852±0.0028	0.3861±0.0024	0.7551±0.0018	0.3390±0.0040
Ours (ResNet)	x	x	0.6875±0.0022	0.3942±0.0019	0.7577±0.0023	0.3447±0.0044
Ours (FT-Trans)	x	x	**0.6963±0.0053**		**0.7693±0.0038**	
BioBERT as text encoder						
Text encoder only		x	0.5927±0.0044	0.2836±0.0037	0.6322±0.0038	0.2114±0.0014
Multimodal	x	x	0.6869±0.0029	0.3712±0.0021	0.7529±0.0027	0.3326±0.0033
Ours (MLP)	x	x	0.6840±0.0033	0.3894±0.0022	0.7539±0.0023	0.3383±0.0039
Ours (ResNet)	x	x	0.6878±0.0030	0.3941±0.0022	0.7587±0.0023	0.3394±0.0055
Ours (FT-Trans)	x	x	**0.6980±0.0040**	**0.3956±0.0045**	**0.7670±0.0039**	**0.3520±0.0048**
Clinical BERT as text encoder						
Text encoder only		x	0.6321±0.0027	0.3227±0.0016	0.6839±0.0027	0.2606±0.0033
Multimodal	x	x	0.6870±0.0026	0.3780±0.0060	0.7592±0.0018	0.3374±0.0056
Ours (MLP)	x	x	0.6890±0.0017	0.3915±0.0030	0.7591±0.0006	0.3446±0.0047
Ours (ResNet)	x	x	0.6884±0.0033	**0.3981±0.0037**	0.7631±0.0022	0.3483±0.0067
Ours (FT-Trans)	x	x		0.3973±0.0052		**0.3532±0.0055**

The best results among models using the same text encoder are in bold, and the overall best results are shaded in grey

**Table 4:** Comparison of predictive reliability performance on MIMIC-III dataset

Model	Struct.	Notes	Brier$\downarrow$	NLL$\downarrow$
(a) Mortality prediction				
Random Forest	x		0.1894±0.0008	0.5640±0.0015
MLP	x		0.1671±0.0018	0.4935±0.0048
ResNet	x		0.1656±0.0007	0.4921±0.0015
FT-Transformer	x		0.1667±0.0041	0.4910±0.0107
BERT as text encoder				
Text encoder only		x	0.2261±0.0060	0.6423±0.0131
Multimodal	x	x	0.1670±0.0046	0.4957±0.0111
Ours (MLP)	x	x	0.1395±0.0035	0.4183±0.0095
Ours (ResNet)	x	x	0.1430±0.0037	0.4311±0.0099
Ours (FT-Trans)	x	x	**0.1366±0.0061**	**0.4125±0.0155**
BioBERT as text encoder				
Text encoder only		x	0.2220±0.0046	0.6328±0.0103
Multimodal	x	x	0.1645±0.0028	0.4919±0.0056
Ours (MLP)	x	x	0.1356±0.0037	0.4091±0.0094
Ours (ResNet)	x	x	0.1523±0.0053	0.4563±0.0138
Ours (FT-Trans)	x	x		
Clinical BERT as text encoder				
Text encoder only		x	0.2091±0.0044	0.5997±0.0107
Multimodal	x	x	0.1637±0.0040	0.4883±0.0092
Ours (MLP)	x	x	**0.1371±0.0067**	**0.4130±0.0179**
Ours (ResNet)	x	x	0.1452±0.0051	0.4379±0.0138
Ours (FT-Trans)	x	x	0.1401±0.0045	0.4198±0.0109
(b) PLOS prediction				
Random Forest	x		0.2066±0.0009	0.6044±0.0019
MLP	x		0.1967±0.0010	0.5777±0.0023
ResNet	x		0.1954±0.0023	0.5746±0.0054
FT-Transformer	x		0.2012±0.0027	0.5846±0.0064
BERT as text encoder				
Text encoder only		x	0.2285±0.0055	0.6491±0.0119
Multimodal	x	x	0.2027±0.0032	0.5969±0.0083
Ours (MLP)	x	x	0.1790±0.0017	0.5321±0.0048
Ours (ResNet)	x	x	0.1736±0.0038	0.5215±0.0095
Ours (FT-Trans)	x	x		
BioBERT as text encoder				
Text encoder only		x	0.2387±0.0028	0.6719±0.0062
Multimodal	x	x	0.2048±0.0049	0.5969±0.0115
Ours (MLP)	x	x	0.1721±0.0031	0.5160±0.0070
Ours (ResNet)	x	x	0.1744±0.0037	0.5235±0.0093
Ours (FT-Trans)	x	x	**0.1704±0.0032**	**0.5026±0.0080**
Clinical BERT as text encoder				
Text encoder only		x	0.2193±0.0010	0.6284±0.0024
Multimodal	x	x	0.1973±0.0081	0.5830±0.0175
Ours (MLP)	x	x	0.1770±0.0024	0.5279±0.0060
Ours (ResNet)	x	x	**0.1699±0.0032**	**0.5122±0.0079**
Ours (FT-Trans)	x	x	0.1744±0.0049	0.5137±0.0125

#### Predictive Reliability

Table 4 compares model performance in terms of reliability on the MIMIC-III dataset. In the mortality prediction task, our framework, with FT-Transformer and BioBERT as backbones, achieved the lowest Brier score (0.1341) and NLL (0.4074). This represents substantial improvements over the best baseline models, with reductions of 18.08% in Brier score and 17.12% in NLL. For the PLOS prediction task, the framework achieved a Brier score of 0.1673 and an NLL of 0.4989, corresponding to improvements of 14.38% and 13.17%, respectively, over the best baseline. Similar patterns were observed in the ZICIP dataset, and further details were discussed in Appendix C.

Compared to other baselines, multimodal approaches achieved reliability comparable to single-modality models in the MIMIC-III dataset but lower reliability in the ZICIP dataset. This may be due to reduced robustness of multimodal models when trained on smaller datasets.

**Fig. 3:**
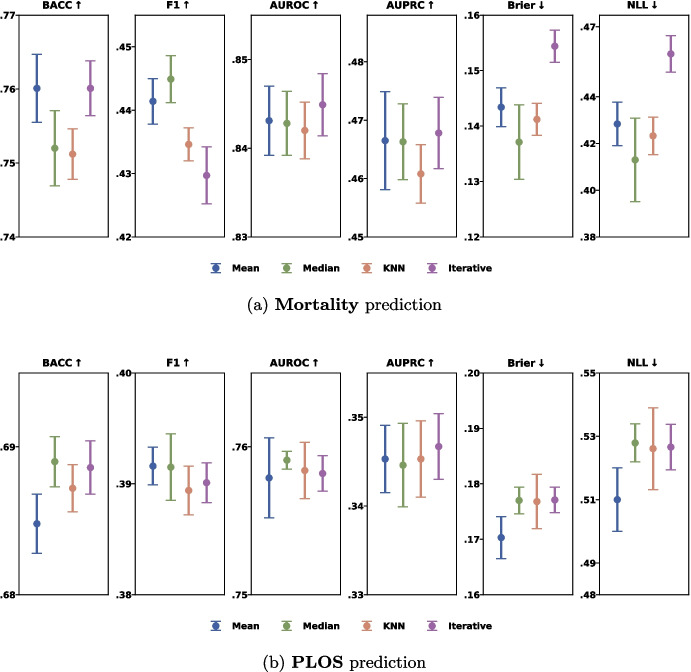
Comparisons of different imputation strategies for missing data: Mean, Median, KNN and Iterative

### Data imputation for missing values

Missing data are common in clinical datasets, and we examined how different imputation strategies for continuous variables affect the performance of our proposed method. Figure 3 compares four imputation techniques–Mean, Median, KNN [49], and Iterative [50] imputations–in MIMIC-III dataset. Overall, the choice of imputation method does not significantly impact the performance of our model. However, we observe that Iterative imputation yields relatively high AURPC but with lower reliability, particularly in mortality prediction. This may be because Iterative imputation relies on predictive models (e.g., Bayesian Ridge, Decision Trees) to estimate missing values. While these models can fit the observed data well, they may not adequately represent the uncertainty in the imputation process.

**Fig. 4:**
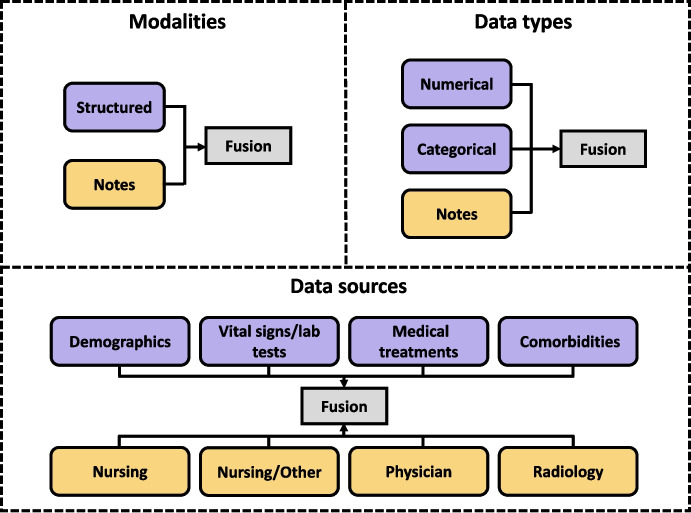
The illustration of different data partitioning settings in MIMIC-III

### Encoder Selection for Data Representation

We further validated the impact of the encoder structure in determining predictability. The evaluations presented in Tables 3 and 8 (Appendix) reveal the comparison results. Among the assessed models, the FT-Transformer stood out as highly effective for structured tabular data, aligning with the findings of [34]. This effectiveness can be attributed to the transformer’s capability to capture complex relationships among transformed numerical and categorical features, which enhances its predictive power.

For pre-trained language encoders, Clinical BERT demonstrated relatively better performance in extracting clinical information from free-text notes in MIMIC-III dataset, likely due to its pre-training on clinical text from the same source. In ZICIP dataset, BERT contributed more to performance improvements in mortality prediction, whereas BioBERT performed better in PLOS prediction.

### Comparisons with other Fusion Strategies

**Fig. 5:**
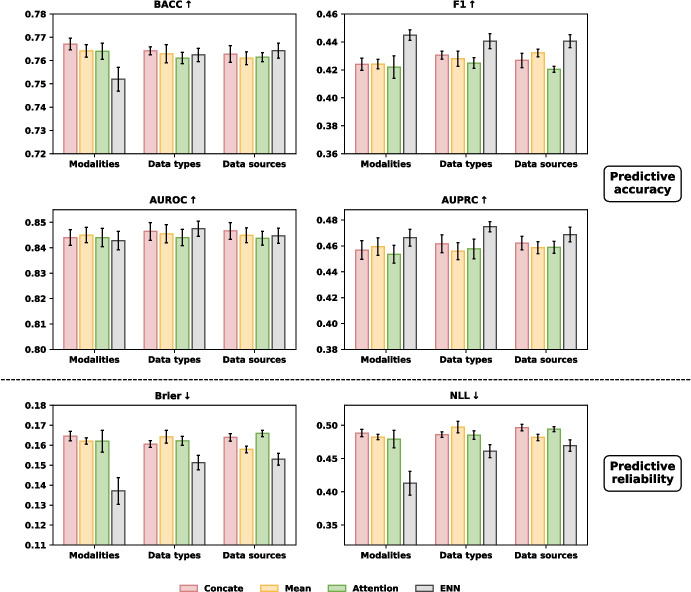
The evaluation of our framework against other fusion strategies on different data partitioning settings for **mortality** prediction: (1) modalities, (2) data types, (3) data sources

**Fig. 6:**
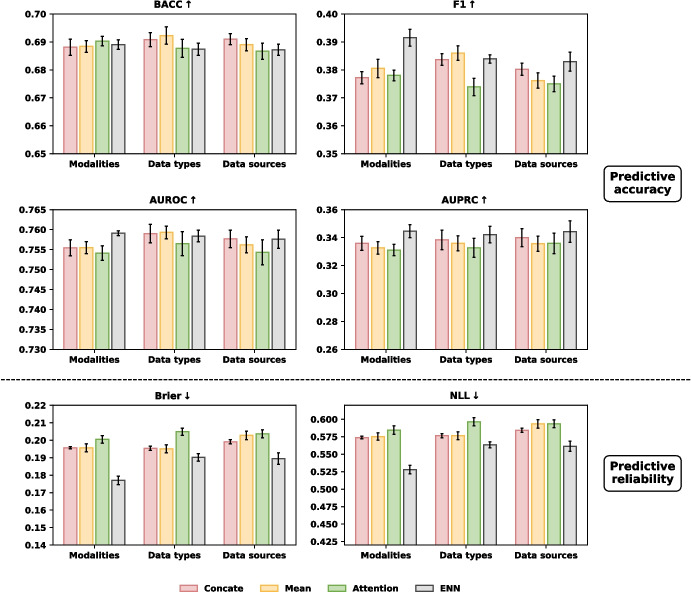
The evaluation of our framework against other fusion strategies on different data partitioning settings for **PLOS** prediction: (1) modalities, (2) data types, (3) data sources

We further examined the effectiveness of our fusion approach by comparing it with other fusion strategies (Concatenation, Mean, and Attention from paper [27]), under three different partitioning settings: data modalities, data types, and data sources. An overview of these settings in MIMIC-III is provided in Fig. 4. The setting of data modalities was previously introduced in the methodology section. For the data types setting, structured data were divided into numerical and categorical subsets. In the data sources setting, structured data were separated into four sources: demographics, vital signs/laboratory tests, medical treatments, and comorbidities. Additionally, clinical notes were grouped into four types: Nursing, Nursing/Other, Physician, and Radiology notes. Figures 5 and 6 present the evaluation results of our framework using the FT-Transformer for structured data and Clinical BERT for notes, across all three data settings.

In terms of predictive accuracy, our proposed fusion strategy generally outperformed alternative fusion methods across all settings, achieving superior results in F1 score and AUPRC. For predictive reliability, it consistently outperformed the other fusion methods in both Brier score and NLL, showing great reliability. The other fusion strategies did not show significant differences in performance over each other.

## Discussion

In this section, we provide further discussion across several key findings: the advantages of using free-text notes, sensitivity to data partitioning settings, the analysis of the evidence-based multimodal framework for ICU outcome prediction, and the interpretability.

### Benefits of Free-Text Notes

From Tables 3 and 8 (Appendix), it is evident that integrating free-text notes with structured data enhances ICU outcome prediction by improving predictive accuracy. Free-text notes capture information not contained in structured EHR data, such as nursing details, physician documentation, and radiology reports after ICU admission. These findings suggest that free-text EHR notes and structured inputs complement each other in predictive modeling, thereby contributing to improved performance. Moreover, the quality and completeness of free-text notes may have influenced model performance. The greater improvement observed in the ZICIP dataset can likely be due to the higher quality of the free-text notes and lower levels of missingness compared with the MIMIC-III dataset.

**Fig. 7:**
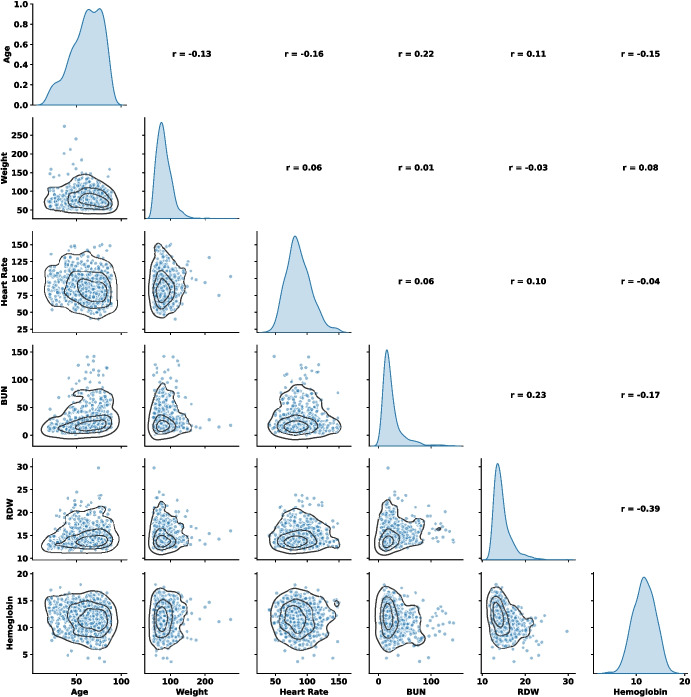
The pair plots and correlation coefficients of some structured features from different sources

**Fig. 8:**
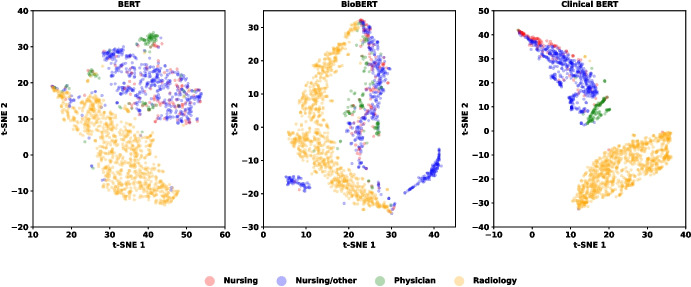
The t-SNE visualization on extracted embeddings of four different types of free-texts in EHRs: Nursing, Nursing/other, Physician, Radiology

### Sensitivity to Data Partition Settings

When applying our proposed fusing strategy on different data partitioning settings (Figs. 5 and 6), modality-level fusion yielded better reliability, as reflected in lower Brier and NLL scores, than fusion based on data types or data sources. This improvement is likely attributable to the more effective integration of information from independent sources enabled by belief function theory. This finding is consistent with our analysis of data independence. Figures 7 and 8 illustrate the independence of structured features and four categories of free-text notes, respectively. As shown in Fig. 7, correlation coefficients indicate that features within structured data are not independent. Similarly, Fig. 8 reveals that radiology notes form a distinct cluster, while the remaining note types overlap substantially, suggesting limited independence among them. It suggests that DST-based modality fusion is a more effective approach in practice, as it provides higher reliability than approaches based on data types or data sources, while maintaining comparable predictive performance.

**Table 5:** Comparison of class-specific prediction accuracy on MIMIC-III dataset

Model	Struct.	Notes	Precision$\uparrow$	Recall$\uparrow$	Specificity$\uparrow$	NPV$\uparrow$
(a) Mortlity prediction						
Random Forest	x		0.2568±0.0035	0.6954±0.0082	0.7304±0.0054	0.9470±0.0011
MLP	x		0.2728±0.0026	0.7753±0.0048	0.7233±0.0047	0.9601±0.0007
ResNet	x		0.2820±0.0021	0.7656±0.0030	0.7391±0.0017	0.9593±0.0006
FT-Transformer	x		0.2825±0.0053	0.7888±0.0073	0.7338±0.0087	0.9629±0.0010
BERT as text encoder						
Text encoder only		x	0.1809±0.0034	0.6599±0.0255	0.6183±0.0290	0.9297±0.0023
Multimodal	x	x	0.2798±0.0064	**0.7908±0.0085**	0.7266±0.0110	**0.9629±0.0010**
Ours (MLP)	x	x	0.3175±0.0061	0.7018±0.0135	0.7976±0.0074	0.9524±0.0018
Ours (ResNet)	x	x	0.3159±0.0055	0.7033±0.0142	0.7954±0.0091	0.9526±0.0017
Ours (FT-Trans)	x	x		0.6998±0.0230		0.9530±0.0026
BioBERT as text encoder						
Text encoder only		x	0.1793±0.0029	0.6535±0.0100	0.5994±0.0087	0.9282±0.0017
Multimodal	x	x	0.2840±0.0050	**0.7859±0.0070**	0.7343±0.0079	**0.9625±0.0009**
Ours (MLP)	x	x	0.3254±0.0077	0.6894±0.0100	**0.8080±0.0083**	0.9511±0.0013
Ours (ResNet)	x	x	0.3039±0.0093	0.7306±0.0077	0.7747±0.0115	0.9555±0.0010
Ours (FT-Trans)	x	x	**0.3319±0.0088**	0.7084±0.0133	0.8079±0.0102	0.9540±0.0014
Clinical BERT as text encoder						
Text encoder only		x	0.1978±0.0030	0.6802±0.0133	0.6308±0.0076	0.9365±0.0021
Multimodal	x	x	0.2896±0.0062		0.7389±0.0104	
Ours (MLP)	x	x	**0.3275±0.0095**	0.7015±0.0257	**0.8052±0.0147**	0.9530±0.0031
Ours (ResNet)	x	x	0.3186±0.0081	0.7313±0.0173	0.7895±0.0113	0.9565±0.0022
Ours (FT-Trans)	x	x	0.3237±0.0089	0.7401±0.0120	0.7916±0.0122	0.9580±0.0014
(b) PLOS prediction						
Random Forest	x		0.2750±0.0025	0.5926±0.0100	0.7546±0.0021	0.9218±0.0015
MLP	x		0.2617±0.0009	0.6594±0.0037	0.7079±0.0022	0.9298±0.0006
ResNet	x		0.2648±0.0036	0.6563±0.0078	0.7135±0.0073	0.9297±0.0011
FT-Transformer	x		0.2579±0.0025	0.7182±0.0137	0.6752±0.0092	0.9386±0.0021
BERT as text encoder						
Text encoder only		x	0.2015±0.0042	0.5519±0.0268	0.6743±0.0170	0.9032±0.0022
Multimodal	x	x	0.2616±0.0040	**0.6810±0.0149**	0.6969±0.0132	**0.9331±0.0018**
Ours (MLP)	x	x	0.2803±0.0014	0.6205±0.0073	0.7498±0.0024	0.9264±0.0011
Ours (ResNet)	x	x	0.2928±0.0045	0.6050±0.0133	**0.7699±0.0097**	0.9256±0.0015
Ours (FT-Trans)	x	x	**0.2940±0.0047**	0.6305±0.0103	0.7622±0.0040	0.9293±0.0018
BioBERT as text encoder						
Text encoder only		x	0.1891±0.0025	0.5676±0.0138	0.6178±0.0095	0.9011±0.0020
Multimodal	x	x	0.2532±0.0031		0.6765±0.0120	
Ours (MLP)	x	x	0.2882±0.0025	0.6017±0.0131	0.7664±0.0072	0.9246±0.0016
Ours (ResNet)	x	x	**0.2923±0.0033**	0.6067±0.0129	**0.7689±0.0079**	0.9257±0.0016
Ours (FT-Trans)	x	x	0.2831±0.0053	0.6584±0.0148	0.7375±0.0110	0.9323±0.0020
Clinical BERT as text encoder						
Text encoder only		x	0.2215±0.0010	0.5897±0.0105	0.6746±0.0057	0.9128±0.0014
Multimodal	x	x	0.2645±0.0088	0.6697±0.0216	0.7044±0.0223	0.9317±0.0023
Ours (MLP)	x	x	0.2857±0.0041	0.6230±0.0065	0.7550±0.0068	0.9273±0.0007
Ours (ResNet)	x	x		0.5975±0.0144		0.9251±0.0017
Ours (FT-Trans)	x	x	0.2822±0.0065	**0.6736±0.0102**	0.7299±0.0119	**0.9344±0.0011**

### Effectiveness of Multimodal Learning Framework

The proposed framework outperformed existing multimodal approaches by leveraging belief function theory to effectively fuse structured and unstructured EHR data. This integration enables more effective, i.e., accurate and robust, predictions, which are essential for clinical decision-making in ICU settings. Although the improvements in BACC are relatively modest (e.g., 0.75% for mortality and 0.72% for PLOS prediction), the gains in F1 score are substantial (6.51% for mortality and 5.70% for PLOS prediction). This underscores the framework’s strength in identifying critical ICU cases, as the F1 score emphasizes both precision and recall. Such improvements are particularly valuable in imbalanced datasets where positive cases are rare. Given the high-stakes nature of ICU outcomes, this suggests that our model can enhance the early identification of high-risk patients and support more timely interventions in critical care. Furthermore, in the context of imbalanced datasets, AUPRC provides a more informative evaluation metric than AUROC. The larger improvements observed in AUPRC across both datasets further demonstrate the framework’s capability to identify high-risk patients under real-world class imbalance.

**Fig. 9:**
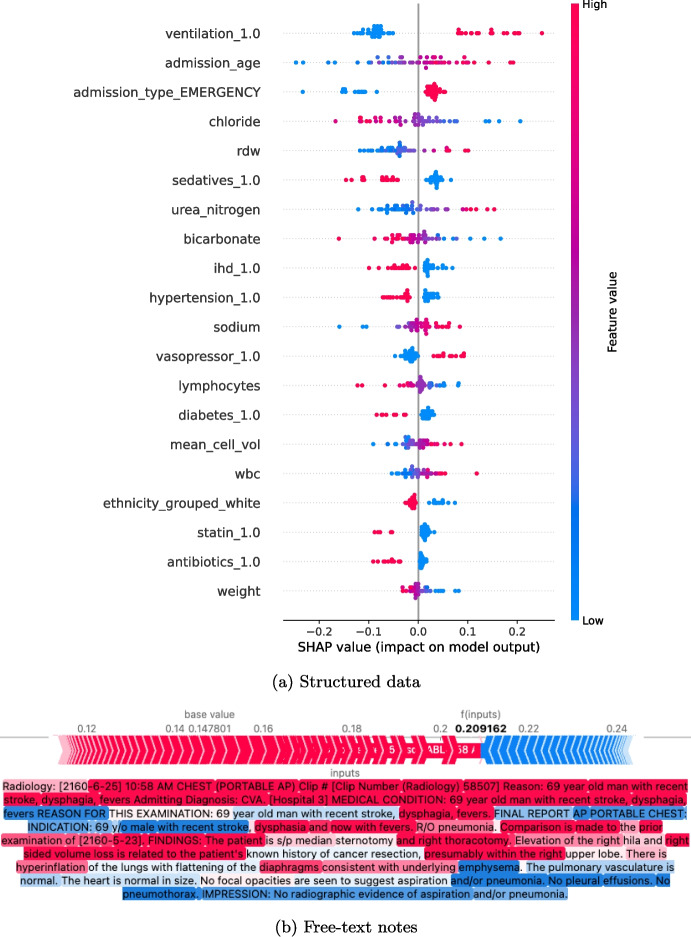
Shapley analysis on structured data and free-text notes

By effectively handling uncertainty and inconsistencies in patient data under the proposed multimodal fusion framework, our approach ensures more trustworthy predictions with lower Brier and NLL (shown in Tables 4 and 9). This enhanced reliability is crucial for ICU decision support, where inaccurate predictions can lead to overuse of critical resources or missed early interventions.

The experimental results in Tables 5 and 12 (Appendix) show that our proposed models achieved higher precision and specificity compared with existing multimodal approaches. This indicates that while conventional multimodal methods are effective in identifying true positive cases, they often come at the cost of increased false positives. In ICU settings, such false positives can lead to the unnecessary use of medical resources and equipment. In contrast, our framework achieves a better balance by demonstrating higher precision and specificity, effectively reducing false positives. This capability is crucial for ensuring that ICU resources are allocated appropriately to patients in critical need.

### Prediction Interpretability

To enhance the interpretability of our proposed approach, we conducted Shapley value analysis separately on the structured data and free-text notes, on the mortality prediction task (MIMIC-III), as illustrated in Fig. 9.

In Fig. 9a, features such as ventilation status, vasopressor use, emergency admission, and older age at admission were associated with higher predicted mortality. On the other hand, the use of antibiotics, statins, and sedatives showed negative SHAP values, suggesting a lower predicted risk of death. This may reflect effective treatment or patient management. Lower body weight was also linked to higher mortality risk, possibly indicating frailty or poor nutritional status. Figure 9b highlights that clinical terms such as “recent stroke,” “dysphagia”, “fevers”, and “hyperinflation” had strong positive SHAP values, consistent with their roles as risk factors for aspiration or lung complications. In contrast, phrases such as “no pleural effusions,” “no pneumothorax,” and “no radiographic evidence of aspiration” showed negative contributions, indicating lower risk due to the absence of concerning findings. These results suggest that the model captures meaningful clinical language, improving its transparency and supporting its potential for trustworthy medical decision-making.

## Conclusion

In this paper, we address the challenge of accurately and reliably predicting ICU outcomes by introducing a multimodal framework based on belief function theory that models both structured EHR data and free-text EHR notes. Our framework transforms deep features extracted from these two modalities into evidence through the evidence mapping module, which is then fused using Dempster’s rule to make final predictions. Through experiments on the MIMIC-III and ZICIP datasets, we demonstrate the effectiveness of our framework in terms of predictive accuracy and reliability. The study highlights its capability in managing heterogeneous multimodal EHR data, reducing false positives, and potentially improving the allocation of medical resources in the ICU.

While this paper focuses on binary classification tasks, many clinical applications require solutions for multiclass tasks (e.g., disease diagnosis) and continuous regression tasks (e.g., survival prediction). These are equally important and relevant for advancing clinical practice.

In the future, we plan to expand our framework by incorporating additional data modalities, such as time series [51–54] and medical images, to provide deeper clinical insights and enhance model performance.

We also aim to extend the framework to handle multimodal EHR multiclass tasks, offering valuable predictive guidance for complex clinical scenarios (Table 6). Additionally, we intend to investigate regression tasks, leveraging the recently introduced Epistemic Random Fuzzy Set (ERFS) theory [26, 55] and further building on developments in evidential regression [24].

## Data Availability

The MIMIC-III dataset used in this study is available through the PhysioNet repository at https://physionet.org/content/mimiciii/1.4/, while the ZICIP dataset can be accessed at https://physionet.org/content/icu-infection-zigong-fourth/1.1/. Access to the datasets is granted only to those who meet the credentialing criteria and accept the terms of use.

## Appendix A: Acronyms

**Table 6:** Acronyms

Acronym	Full term
MIMIC-III	Medical Information Mart for Intensive Care III
ZICIP	Zigong Intensive Care Infection Patients
BFT	Belief function theory
ENN	Evidential neural network
EM	Evidence mapping
EF	Evidence fusion
MLP	Multilayer perceptron
ResNet	Residual neural network
FT-Transformer	Feature tokenizer transformer
FCN	Fully connected network
PLOS	Prolonged length of stay
NPV	Negative predictive value
BACC	Balanced accuracy
AUROC	The area under the receiver operating characteristic curve
AUPRC	The area under the precision-recall curve
NLL	Negative log-likelihood

## Appendix B: Patient Characteristics of ZICIP Dataset

**Table 7:** Characteristics of structured features in the ZICIP patient cohort

Patient	Mortality		PLOS	
characteristic				
	Yes (N=161)	No (N=2629)	Yes (N=965)	No (N=1825)
Age	66.7 (20.0)	66.6 (18.2)	67.3 (17.5)	66.2 (18.7)
Gender				
Male	112 (69.6%)	1564 (59.5%)	631 (65.4%)	1045 (57.3%)
Female	49 (30.4%)	1065 (40.5%)	334 (34.6%)	780 (42.7%)
Temperature	37.0 (1.1)	36.8 (0.7)	36.8 (0.7)	36.8 (0.7)
Heart rate	103.7 (28.0)	96.8 (26.1)	95.6 (26.0)	97.9 (26.3)
$\text {SpO}_{2}$	92.2 (17.2)	95.6 (12.5)	96.5 (9.2)	95.0 (13.9)
SBP	114.8 (37.8)	127.0 (31.5)	127.1 (29.1)	126.0 (33.1)
APTT	39.1 (21.1)	30.9 (10.4)	30.7 (9.8)	31.7 (12.2)
EOS#	0.1 (0.2)	0.1 (0.1)	0.1 (0.1)	0.1 (0.1)
LYM#	77.3 (17.7)	76.6 (13.9)	77.8 (15.2)	76.5 (14.4)
NEU#	11.8 (7.2)	10.9 (6.5)	9.9 (6.1)	11.5 (6.7)
RDWSD	46.6 (5.6)	47.1 (6.3)	47.0 (6.1)	47.2 (6.4)
CRE	112.6 (107.6)	107.1 (133.5)	102.3 (114.9)	110.2 (140.3)
Hgb	116.3 (25.1)	117.5 (27.9)	119.2 (27.4)	116.5 (27.8)
MCV	93.8 (9.9)	92.8 (8.8)	92.8 (8.2)	92.8 (9.2)
PLT	177.1 (79.1)	170.0 (93.4)	168.7 (80.1)	171.4 (98.5)
Potassium	3.8 (1.0)	3.7 (0.8)	3.7 (0.7)	3.7 (0.8)
Sodium	137.5 (8.6)	138.1 (5.5)	138.5 (4.8)	137.9 (6.1)
PT	18.8 (13.3)	14.8 (4.5)	14.5 (4.2)	15.3 (6.0)
WBC	14.4 (8.2)	12.6 (7.0)	11.6 (6.5)	13.3 (7.3)
PH	7.3 (0.2)	7.4 (0.1)	7.4 (0.1)	7.3 (0.1)
Urea	9.3 (6.9)	9.3 (7.6)	9.0 (7.9)	9.4 (7.4)
Calcium	2.2 (0.2)	2.2 (0.2)	2.2 (0.2)	2.2 (0.2)
$\text {CO}_{2}$	20.6 (8.1)	22.2 (6.3)	23.1 (5.9)	21.6 (6.6)
Albumin	32.7 (7.1)	34.5 (7.1)	34.7 (7.0)	34.3 (7.2)
Ventilation	43 (32.1%)	755 (37.5%)	289 (49.6%)	509 (32.5%)
Antibiotic	15 (10.5%)	374 (18.3%)	133 (19.1%)	256 (17.2%)
Diuretic	62 (43.4%)	981 (48.0%)	338 (48.5%)	705 (47.3%)
Sedative	56 (39.2%)	615 (30.1%)	187 (26.8%)	484 (32.5%)
Statin	19 (13.3%)	349 (17.1%)	119 (17.1%)	249 (16.7%)
Vasopressor	55 (38.5%)	717 (35.1%)	225 (32.3%)	580 (39.0%)
Alcohol abuse	3 (3.2%)	46 (3.0%)	9 (1.6%)	40 (3.9%)
CVA	30 (32.3%)	483 (31.9%)	201 (34.7%)	312 (30.4%)
CHF	2 (2.2%)	121 (8.0%)	52 (8.0%)	71 (6.9%)
Diabetes	32 (34.4%)	496 (32.8%)	206 (35.5%)	322 (31.4%)
Hypertension	41 (44.1%)	515 (34.0%)	189 (32.6%)	367 (35.7%)
IHD	22 (23.7%)	311 (20.6%)	117 (20.2%)	216 (21.0%)

Table 7 presents the characteristics of patients in the ZICIP dataset. The dataset includes 2,790 patients with infections, of whom 161 (5.8%) died and 965 (34.6%) experienced prolonged ICU stays. Male patients outnumbered female patients. Those who died generally showed higher heart rates and activated partial thromboplastin time (APTT). The most common comorbidities were cerebrovascular accident (CVA), diabetes, hypertension, and ischemic heart disease (IHD).

## Appendix C: Evaluation results on ZICIP dataset in predictive accuracy and reliability

**Table 8:** Comparison of predictive accuracy on ZICIP dataset

Model	Struct.	Notes	BACC$\uparrow$	F1$\uparrow$	AUROC$\uparrow$	AUPRC$\uparrow$
(a) Mortality prediction						
Random Forest	x		0.5623±0.0128	0.1592±0.0170	0.6423±0.0186	0.1445±0.0185
MLP	x		0.5959±0.0125	0.1750±0.0125	0.6289±0.0185	0.1589±0.0154
ResNet	x		0.6015±0.0148	0.1714±0.0114	0.6400±0.0102	0.1328±0.0071
FT-Transformer	x		0.6225±0.0070	0.1805±0.0107	0.6681±0.0166	0.1829±0.0181
BERT as text encoder						
Text encoder only		x	0.5658±0.0094	0.1345±0.0053	0.5696±0.0244	0.0980±0.0113
Multimodal	x	x	0.6144±0.0182	0.1658±0.0139	0.6548±0.0237	0.1613±0.0145
Ours (MLP)	x	x	0.5942±0.0130	0.2178±0.0233	0.6567±0.0159	0.2031±0.0136
Ours (ResNet)	x	x	0.5944±0.0132	0.2017±0.0201	0.6213±0.0125	0.1913±0.0107
Ours (FT-Trans)	x	x				
BioBERT as text encoder						
Text encoder only		x	0.5647±0.0105	0.1319±0.0051	0.5875±0.0139	0.0982±0.0096
Multimodal	x	x	0.5966±0.0127	0.1520±0.0080	0.6452±0.0208	0.1843±0.0159
Ours (MLP)	x	x	0.5998±0.0125	0.2131±0.0222	0.6499±0.0180	0.2028±0.0093
Ours (ResNet)	x	x	0.5720±0.0136	0.1693±0.0180	0.6296±0.0125	0.1857±0.0099
Ours (FT-Trans)	x	x	**0.6133±0.0040**	**0.2273±0.0134**	**0.6683±0.0101**	**0.2338±0.0170**
Clinical BERT as text encoder						
Text encoder only		x	0.5500±0.0175	0.1278±0.0093	0.5684±0.0240	0.0946±0.0108
Multimodal	x	x	0.6015±0.0122	0.1597±0.0112	0.6388±0.0211	0.1753±0.0180
Ours (MLP)	x	x	0.5852±0.0104	0.1930±0.0086	0.6606±0.0100	0.2150±0.0118
Ours (ResNet)	x	x	0.5863±0.0071	0.1900±0.0088	0.6459±0.0180	0.1860±0.0104
Ours (FT-Trans)	x	x	**0.6119±0.0073**	**0.2235±0.0150**	**0.6704±0.0090**	**0.2254±0.0154**
(b) PLOS prediction						
Random Forest	x		0.6130±0.0094	0.4976±0.0141	0.6543±0.0141	0.5226±0.0136
MLP	x		0.5820±0.0086	0.4899±0.0077	0.6227±0.008	0.4905±0.0102
ResNet	x		0.5907±0.0061	0.5058±0.0082	0.6321±0.0066	0.4828±0.0044
FT-Transformer	x		0.6137±0.0106	0.5329±0.0141	0.6729±0.0103	0.5400±0.0158
BERT as text encoder						
Text encoder only		x	0.6304±0.0074	0.5344±0.0109	0.6793±0.0104	0.5143±0.0138
Multimodal	x	x	0.6247±0.0084	0.5452±0.0107	0.6850±0.0094	0.5561±0.0151
Ours (MLP)	x	x	0.6349±0.0074	0.5414±0.0113	0.6854±0.0113	0.5753±0.0185
Ours (ResNet)	x	x	**0.6452±0.0089**	0.5436±0.0098	0.6875±0.0082	0.5752±0.0126
Ours (FT-Trans)	x	x	0.6321±0.0087	**0.5461±0.0073**	**0.6911±0.0106**	**0.5762±0.0132**
BioBERT as text encoder						
Text encoder only		x	0.6306±0.0072	0.5525±0.0115	0.6815±0.0114	0.5141±0.0130
Multimodal	x	x	0.6518±0.0058		0.6974±0.0076	0.5604±0.0116
Ours (MLP)	x	x	0.6434±0.0106	0.5414±0.0120		**0.5892±0.0127**
Ours (ResNet)	x	x		0.5566±0.0105	0.7052±0.0101	0.5866±0.0146
Ours (FT-Trans)	x	x	0.6346±0.0093	0.5479±0.0062	0.6940±0.0101	0.5743±0.0135
Clinical BERT as text encoder						
Text encoder only		x	0.6305±0.0097	**0.5539±0.0126**	0.6868±0.0129	0.5185±0.0157
Multimodal	x	x	0.6288±0.0044	0.5471±0.0114	0.6951±0.0109	0.5703±0.0176
Ours (MLP)	x	x	0.6427±0.0110	0.5352±0.0142	0.7025±0.0095	0.5865±0.0183
Ours (ResNet)	x	x	**0.6532±0.0116**	0.5520±0.0186	**0.7034±0.0117**	
Ours (FT-Trans)	x	x	0.6334±0.0095	0.5536±0.0077	0.6916±0.0099	0.5792±0.0146

**Table 9:** Comparison of predictive reliability performance on ZICIP dataset

Model	Struct.	Notes	Brier$\downarrow$	NLL$\downarrow$
(a) Mortality prediction				
Random Forest	x		0.1454±0.0029	0.4700±0.0057
MLP	x		0.1937±0.0057	0.5803±0.0134
ResNet	x		0.1968±0.0031	0.5878±0.0084
FT-Transformer	x		0.1962±0.0126	0.5814±0.0271
BERT as text encoder				
Text encoder only		x	0.2467±0.0107	0.6898±0.0229
Multimodal	x	x	0.2327±0.0157	0.6710±0.0319
Ours (MLP)	x	x	0.1136±0.0028	0.3903±0.0086
Ours (ResNet)	x	x	0.1110±0.0059	0.3774±0.0170
Ours (FT-Trans)	x	x		
BioBERT as text encoder				
Text encoder only		x	0.2369±0.0058	0.6687±0.0122
Multimodal	x	x	0.2221±0.0095	0.6464±0.0211
Ours (MLP)	x	x	0.1093±0.0041	**0.3700±0.0145**
Ours (ResNet)	x	x	0.1123±0.0048	0.3778±0.0150
Ours (FT-Trans)	x	x	**0.1058±0.0069**	0.3707±0.0126
Clinical BERT as text encoder				
Text encoder only		x	0.2395±0.0103	0.6737±0.0215
Multimodal	x	x	0.2170±0.0135	0.6350±0.0280
Ours (MLP)	x	x	0.1049±0.0069	0.3627±0.0178
Ours (ResNet)	x	x	**0.1044±0.0035**	**0.3612±0.0103**
Ours (FT-Trans)	x	x	0.1048±0.0054	0.3635±0.0102
(b) PLOS prediction				
Random Forest	x		0.2258±0.0024	0.6433±0.0050
MLP	x		0.2390±0.0014	0.6706±0.0032
ResNet	x		0.2393±0.0009	0.6716±0.0023
FT-Transformer	x		0.2397±0.0026	0.6746±0.0064
BERT as text encoder				
Text encoder only		x	0.2307±0.0026	0.6548±0.0057
Multimodal	x	x	0.2354±0.0010	0.6650±0.0018
Ours (MLP)	x	x	0.2247±0.0024	0.6461±0.0052
Ours (ResNet)	x	x	**0.2181±0.0048**	**0.6323±0.0140**
Ours (FT-Trans)	x	x	0.2308±0.0057	0.6554±0.0120
BioBERT as text encoder				
Text encoder only		x	0.2459±0.0044	0.6917±0.0105
Multimodal	x	x	0.2272±0.0042	0.6543±0.0089
Ours (MLP)	x	x	0.2136±0.0085	0.6207±0.0169
Ours (ResNet)	x	x		
Ours (FT-Trans)	x	x	0.2282±0.0081	0.6496±0.0182
Clinical BERT as text encoder				
Text encoder only		x	0.2458±0.0044	0.6919±0.0108
Multimodal	x	x	0.2342±0.0010	0.6640±0.0026
Ours (MLP)	x	x	**0.2144±0.0036**	**0.6194±0.0074**
Ours (ResNet)	x	x	0.2149±0.0063	0.6251±0.0144
Ours (FT-Trans)	x	x	0.2337±0.0053	0.6605±0.0114

Table 8 reports the results on the ZICIP dataset. In the mortality prediction task, our framework with FT-Transformer and BERT outperformed the baselines, achieving a BACC of 0.6241, F1 score of 0.2400, AUROC of 0.6735, and AUPRC of 0.2360. Compared with the best baselines, this represents gains of 0.26% in BACC, 32.96% in F1 score, 0.81% in AUROC, and 28.05% in AUPRC. In the PLOS prediction task, our model achieved the highest BACC (0.6551), AUROC (0.7064), and AUPRC (0.5897), with improvements of 0.51%, 1.29%, and 3.40%, respectively, over the best baseline models.

Table 9 presents the reliability results on the ZICIP dataset. In the mortality prediction task, our framework obtained the lowest Brier score (0.0978) and NLL (0.3421), with reductions of 32.74% and 27.21%, respectively, compared with the best baseline models. In the PLOS prediction task, it achieved a Brier score of 0.2120 and NLL of 0.6156, yielding improvements of 6.11% and 4.31%, respectively.

## Appendix D: T-test Matrices of Various Model Performances (AUPRC)

**Table 10:** P-value performance matrices on MIMIC-III dataset

		Struct. only				Notes only			Both	
		RF	MLP	ResNet	FT	BERT	BioBERT	C-BERT	MM	Ours
(a) Mortality prediction										
Struct. only	RF	1	<0.050	<0.050	<0.050	<0.050	<0.050	<0.050	<0.050	<0.050
	MLP	<0.050	1	<0.050	<0.050	<0.050	<0.050	<0.050	<0.050	<0.050
	ResNet	<0.050	<0.050	1	0.053	<0.050	<0.050	<0.050	<0.050	<0.050
	FT	<0.050	<0.050	0.053	1	<0.050	<0.050	<0.050	0.482	<0.050
Notes only	BERT	<0.050	<0.050	<0.050	<0.050	1	0.105	<0.050	<0.050	<0.050
	BioBERT	<0.050	<0.050	<0.050	<0.050	0.105	1	<0.050	<0.050	<0.050
	C-Bert	<0.050	<0.050	<0.050	<0.050	<0.050	<0.050	1	<0.050	<0.050
Both	MM	<0.050	<0.050	<0.050	0.482	<0.050	<0.050	<0.050	1	<0.050
	Ours	<0.050	<0.050	<0.050	<0.050	<0.050	<0.050	<0.050	<0.050	1
(b) PLOS prediction.										
Struct. only	RF	1	<0.050	<0.050	<0.050	<0.050	<0.050	<0.050	<0.050	<0.050
	MLP	<0.050	1	0.396	<0.050	<0.050	<0.050	<0.050	0.369	<0.050
	ResNet	<0.050	<0.050	1	<0.050	<0.050	<0.050	<0.050	0.247	<0.050
	FT	<0.050	<0.050	<0.050	1	<0.050	<0.050	<0.050	0.447	<0.050
Notes only	BERT	<0.050	<0.050	<0.050	<0.050	1	0.152	<0.050	<0.050	<0.050
	BioBERT	<0.050	<0.050	<0.050	<0.050	0.152	1	<0.050	<0.050	<0.050
	C-Bert	<0.050	<0.050	<0.050	<0.050	<0.050	<0.050	1	<0.050	<0.050
Both	MM	<0.050	0.369	0.247	0.447	<0.050	<0.050	<0.050	1	<0.050
	Ours	<0.050	<0.050	<0.050	<0.050	<0.050	<0.050	<0.050	<0.050	1

**Table 11:** P-value performance matrices on ZICIP dataset

		Struct. only				Notes only			Both	
		RF	MLP	ResNet	FT	BERT	BioBERT	C-BERT	MM	Ours
(a) Mortality prediction										
Struct. only	RF	1	0.211	0.413	0.066	0.105	0.134	<0.050	0.078	<0.050
	MLP	0.211	1	<0.050	<0.050	<0.050	<0.050	<0.050	<0.050	<0.050
	ResNet	0.413	<0.050	1	<0.050	<0.050	0.080	<0.050	<0.050	<0.050
	FT	0.066	<0.050	<0.050	1	<0.050	<0.050	<0.050	0.902	<0.050
Notes only	BERT	0.105	<0.050	<0.050	<0.050	1	0.988	0.864	<0.050	<0.050
	BioBERT	0.134	<0.050	0.080	<0.050	0.988	1	0.867	<0.050	<0.050
	C-Bert	<0.050	<0.050	<0.050	<0.050	0.864	0.867	1	<0.050	<0.050
Both	MM	0.078	<0.050	<0.050	0.902	<0.050	<0.050	<0.050	1	<0.050
	Ours	<0.050	<0.050	<0.050	<0.050	<0.050	<0.050	<0.050	<0.050	1
(b) PLOS prediction										
Struct. only	RF	1	0.211	0.413	0.066	0.105	0.134	<0.050	0.078	<0.050
	MLP	0.211	1	<0.050	<0.050	<0.050	<0.050	<0.050	<0.050	<0.050
	ResNet	0.413	<0.050	1	<0.050	<0.050	0.080	<0.050	<0.050	<0.050
	FT	0.066	<0.050	<0.050	1	<0.050	<0.050	<0.050	0.902	<0.050
Notes only	BERT	0.105	<0.050	<0.050	<0.050	1	0.988	0.864	<0.050	<0.050
	BioBERT	0.134	<0.050	0.080	<0.050	0.988	1	0.867	<0.050	<0.050
	C-Bert	<0.050	<0.050	<0.050	<0.050	0.864	0.867	1	<0.050	<0.050
Both	MM	0.078	<0.050	<0.050	0.902	<0.050	<0.050	<0.050	1	<0.050
	Ours	<0.050	<0.050	<0.050	<0.050	<0.050	<0.050	<0.050	<0.050	1

RF: Random Forest, C-BERT: Clinical BERT, MM: Multimodal

## Appendix E: Evaluation Results on ZICIP Dataset in Class-Specific Prediction Accuracy

**Table 12:** Comparison of class-specific prediction accuracy on ZICIP dataset

Model	Struct.	Notes	Precision$\uparrow$	Recall$\uparrow$	Specificity$\uparrow$	NPV$\uparrow$
(a) Mortality prediction						
Random Forest	x		0.1278±0.0143	0.2178±0.0267	0.9068±0.0103	0.9498±0.0017
MLP	x		0.1154±0.0091	0.3663±0.0221	0.8254±0.0122	0.9551±0.0016
ResNet	x		0.1085±0.0074	0.4104±0.0299	0.7927±0.0116	0.9564±0.0023
FT-Transformer	x		0.1129±0.0094	0.5028±0.0389	0.7421±0.0470	0.9607±0.0008
BERT as text encoder						
Text encoder only		x	0.0785±0.0041	0.4909±0.0368	0.6406±0.0375	0.9536±0.0019
Multimodal	x	x	0.0985±0.0102		0.6637±0.0501	
Ours (MLP)	x	x		0.2604±0.0309		0.9535±0.0014
Ours (ResNet)	x	x	0.1592±0.0192	0.2854±0.0277	0.9034±0.0134	0.9538±0.0015
Ours (FT-Trans)	x	x	0.1875±0.0159	0.3413±0.0266	0.9068±0.0112	0.9574±0.0014
BioBERT as text encoder						
Text encoder only		x	0.0761±0.0030	0.5106±0.0491	0.6197±0.0383	0.9541±0.0023
Multimodal	x	x	0.0891±0.0053	**0.5286±0.0268**	0.6645±0.0264	**0.9583±0.0020**
Ours (MLP)	x	x	0.1708±0.0239	0.2917±0.0200	**0.9079±0.0115**	0.9544±0.0014
Ours (ResNet)	x	x	0.1292±0.0148	0.2487±0.0247	0.8954±0.0087	0.9531±0.0037
Ours (FT-Trans)	x	x	**0.1815±0.0207**	0.3233±0.0172	0.9034±0.0161	0.9562±0.0007
Clinical BERT as text encoder						
Text encoder only		x	0.0749±0.0063	0.4591±0.0380	0.6409±0.0429	0.9506±0.0028
Multimodal	x	x	0.0956±0.0088	**0.5214±0.0360**	0.6816±0.0461	**0.9588±0.0013**
Ours (MLP)	x	x	0.1680±0.0127	0.2542±0.0376	**0.9163±0.0200**	0.9526±0.0014
Ours (ResNet)	x	x	0.1486±0.0079	0.2669±0.0177	0.9056±0.0067	0.9528±0.0008
Ours (FT-Trans)	x	x	**0.1766±0.0202**	0.3227±0.0223	0.9011±0.0162	0.9560±0.0008
(b) PLOS prediction						
Random Forest	x		0.4869±0.0100	0.5098±0.0209	0.7161±0.01	0.7346±0.0075
MLP	x		0.4252±0.0094	0.5782±0.0048	0.5868±0.0131	0.7239±0.0065
ResNet	x		0.4277±0.0048	0.6209±0.0174	0.5616±0.0106	0.7369±0.0070
FT-Transformer	x		0.4455±0.0097	0.6674±0.0351	0.5600±0.0267	0.7634±0.0132
BERT as text encoder						
Text encoder only		x	0.4863±0.0124	0.6000±0.0326	0.6608±0.0324	0.7587±0.0075
Multimodal	x	x	0.4548±0.0076	**0.6829±0.0257**	0.5666±0.0190	**0.7728±0.0101**
Ours (MLP)	x	x	0.4859±0.0083	0.6155±0.0302	0.6542±0.0219	0.7643±0.0096
Ours (ResNet)	x	x	**0.5208±0.0171**	0.5720±0.0189	**0.7183±0.0234**	0.7606±0.0064
Ours (FT-Trans)	x	x	0.4745±0.0144	0.6477±0.0201	0.6164±0.0299	0.7681±0.0061
BioBERT as text encoder						
Text encoder only		x	0.4595±0.0115	**0.7057±0.0476**	0.5556±0.0436	**0.7863±0.0153**
Multimodal	x	x	0.5000±0.0108	0.6487±0.0151	0.6548±0.0211	0.7791±0.0039
Ours (MLP)	x	x	0.5297±0.0296	0.5751±0.0499	0.7118±0.0615	0.7625±0.0088
Ours (ResNet)	x	x	**0.5297±0.0145**	0.5886±0.0182	**0.7217±0.0200**	0.7686±0.0062
Ours (FT-Trans)	x	x	0.4811±0.0181	0.6446±0.0261	0.6246±0.0400	0.7690±0.0055
Clinical BERT as text encoder						
Text encoder only		x	0.4559±0.0093		0.5480±0.0314	
Multimodal	x	x	0.4615±0.0077	0.6829±0.0441	0.5748±0.0378	0.7779±0.0120
Ours (MLP)	x	x		0.5451±0.0252		0.7551±0.0073
Ours (ResNet)	x	x	0.5296±0.0187	0.5907±0.0514	0.7156±0.0418	0.7715±0.0153
Ours (FT-Trans)	x	x	0.4671±0.0141	0.6850±0.0227	0.5819±0.0333	0.7776±0.0068

## Footnotes

1 This work is an extended version of the short paper presented at the 8th International Conference on Belief Functions (BELIEF 2024) [27].

2 https://scikit-learn.org/stable/modules/generated/sklearn.utils.class_weight.compute_class_weight.html

3 https://github.com/yandex-research/rtdl-revisiting-models.

4 https://github.com/WeiChunLin/Bio_Clinical_BERT_Multimodal_Model.
